# 258. Clinical *Rel* Mutations in *Staphylococcus aureus* Primes Pathogen Expansion Under Nutrient Stress

**DOI:** 10.1093/ofid/ofad500.330

**Published:** 2023-11-27

**Authors:** Edwin Chen, Marla G Shaffer, Robert E Bilodeau, Raymond E West, Patrick J Oberly, Thomas D Nolin, Matthew J Culyba

**Affiliations:** University of Pittsburgh, Pittsburgh, Pennsylvania; University of Pittsburgh, Pittsburgh, Pennsylvania; University of Pittsburgh, Pittsburgh, Pennsylvania; University of PIttsburgh, Pittsburgh, Pennsylvania; University of Pittsburgh, Pittsburgh, Pennsylvania; University of Pittsburgh, Pittsburgh, Pennsylvania; University of PIttsburgh, Pittsburgh, Pennsylvania

## Abstract

**Background:**

Nutritional immunity is an important component of host defense whereby nutrients are sequestered to limit pathogen replication. The bacterial stringent response (SR) enables cells to modulate their growth, metabolism, and virulence in accordance with available nutrition. By analyzing within-host evolution of MRSA in patients with persistent bacteremia, we recently identified 5 protein-altering mutations in *rel*. Rel synthesizes or degrades ‘alarmone’ molecules to regulate the SR in response to stress conditions. Here, we characterized the phenotypes caused by these mutations and linked them to a mechanism for evading nutritional immunity.

Rel domain architecture and location of clinical mutations.

**Figure:**
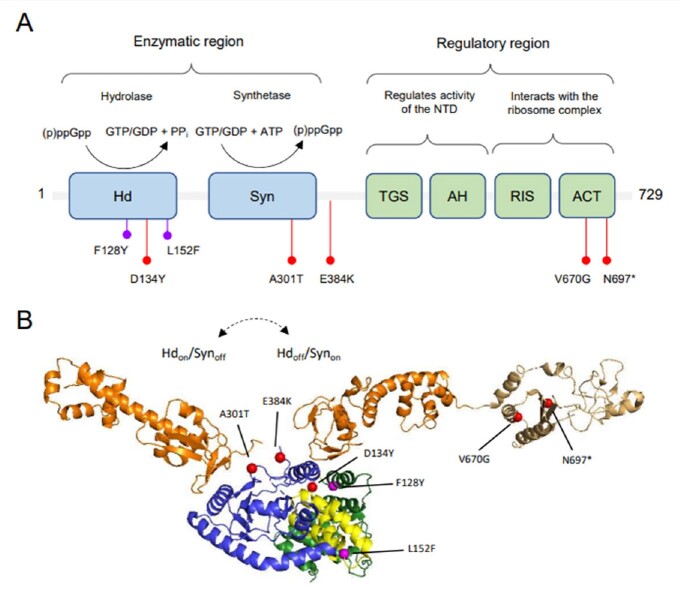

(A) The N-terminal enzymatic region containing the hydrolase (Hd) and synthetase (Syn) domains are shown in blue. The C-terminal regulatory region containing the TGS (ThrRs, GTPase, SpoT), AH (Alpha-Helical), RIS (Ribosome Inter Subunit), and ACT (Aspartate kinase, Chorismate mutase, TyrA) domains are shown in green. The Hd domain catalyzes the hydrolysis of (p)ppGpp to yield inorganic pyrophosphate (PPi) and either GTP or GDP. The Syn domain catalyzes the transfer of pyrophosphate from ATP to either GTP or GDP to form (p)ppGpp. The mutations examined in this study are denoted with red markers. Previously identified mutations are denoted with purple markers. (B) Clinical Rel mutations mapped onto a structure of a Hdon/Synoff RelΔRIS-CT from B. subtilis (PDB 6YXA) with the CTD of a Hdoff/Synon Rel from E. coli (PDB 5KPV) superimposed. Domain colorings: hydrolase, green; central 3-helix bundle, yellow; synthetase, blue; TGS-AH, orange; RIS-ACT, tan. Rel mutations focused in this study are denoted by red spheres. Previously identified mutations are denoted by purple spheres.

**Methods:**

Mutations were introduced into the *rel* locus of MRSA strain JE2 by allelic exchange. The growth, fitness, and antibiotic profiles (vancomycin, daptomycin, ceftaroline, mupirocin) of the mutant strains were compared to wildtype in different nutrient conditions. Mass spectrometry (MS) was used to quantify intracellular alarmone levels during different phases of growth.

**Results:**

We found that the *rel* mutations caused increased SR signaling and conveyed a survival advantage during stationary phase growth. The mutants additionally displayed a fitness advantage when subsequently seeded in nutrient-limiting media. This suggested that the cells become primed for survival and replication during the stationary phase to facilitate pathogen expansion. In contrast to previous studies, we found our mutants do not exhibit an antibiotic tolerance phenotype.

Rel mutants have a growth fitness advantage under nutrient stress.

**Figure:**
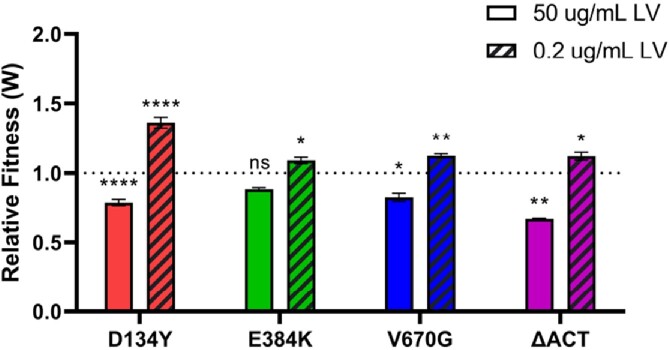

Relative fitness of Rel mutant strains compared to WT, each competing with WT-GFP in CDM containing 50 ug/mL LV or 0.2 ug/mL LV. A relative fitness value of 1.0, marked by the dotted line, denotes equal fitness with WT. Data points and error represents the mean and SEM of three independent biologic replicates, respectively. Mean values were compared with the internal WT/WT-GFP control using unpaired t-test (ns, not significant; *, P ≤ 0.05; **, P ≤ 0.01; ****, P ≤ 0.0001).

Rel mutations are partially activating during stationary phase.

**Figure:**
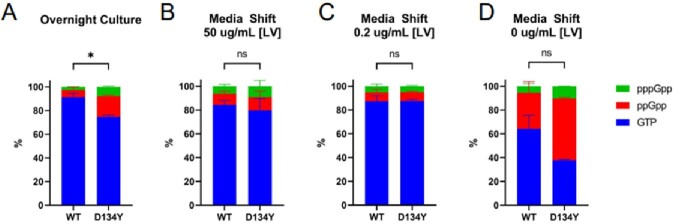

LC-MS/MS quantification of intracellular GTP, ppGpp, and pppGpp of (A) overnight cultures grown in TSB, and overnight cultures grown in TSB subsequently media-shifted into (B) 50 ug/mL LV CDM, (C) 0.2 ug/mL LV CDM, and (D) 0 ug/mL LV CDM. Quantification is displayed as % composition of the quantified guanosine nucleotide pool. Data points and error represents the mean and SEM of three independent biological replicates, respectively. Mean values of % GTP were compared using unpaired t-test (ns, not significant; *, P ≤ 0.05).

Rel mutants have a survival advantage under nutrient stress.

**Figure:**
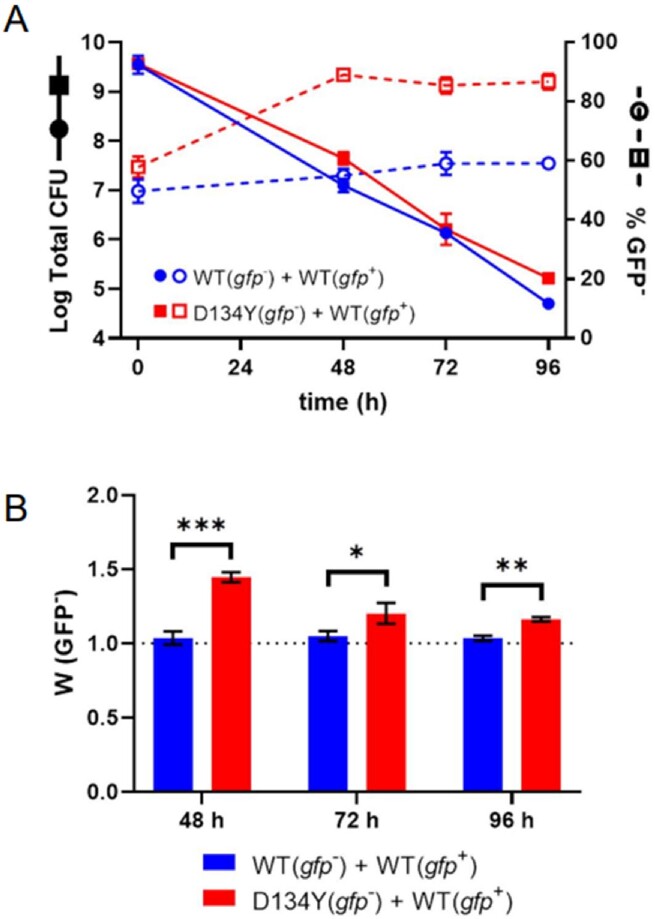

(A) Survival of co-cultures of WT-GFP (gfp+) and either WT or D134Y (gfp-) during continuous stationary phase incubation. Data at each timepoint is represented by total population CFU and % gfp- cells in the surviving population. (B) Survival fitness of WT and D134Y (gfp-) relative to WT-GFP (gfp+) in co-culture at designated timepoints during continuous stationary phase incubation. A survival fitness value of 1.0, marked by the dotted line, denotes no survival advantage nor disadvantage. Data points and error represents the mean and SEM of three independent biological replicates, respectively. Comparisons were performed using unpaired t-test (*, P ≤ 0.05; **, P ≤ 0.01; ***, P ≤ 0.001).

**Conclusion:**

The SR plays a key role at the intersection of metabolism and persistent infection. Our findings show that nutrient stress within host tissues selects for adaptive mutations in bacteria which serve to counteract nutritional immunity. The clinical *rel* mutations we characterized caused a fitness advantage by enhancing alarmone production under stress conditions, thereby augmenting the existing stress-response programming to improve growth and survival. Elucidating the detailed mechanisms involved will provide valuable insight into the development of anti-metabolism therapeutics to both enhance host nutritional immunity and to serve as adjunctive therapies alongside traditional antibiotics.

**Disclosures:**

**All Authors**: No reported disclosures

